# Antiaging Effect of 2-*O*-β-D-Glucopyranosyl Ascorbic Acid Derived from *Lycium barbarum* L. Through Modulating the IIS Pathway and Gut Microbiota in *Caenorhabditis elegans*

**DOI:** 10.3390/foods14111875

**Published:** 2025-05-25

**Authors:** Jiayue Fang, Wei Dong, Jingqian Zheng, Boxuan Han, Yuying Zhang, Jianing Wang, Xiaoxiong Zeng

**Affiliations:** College of Food Science and Technology, Nanjing Agricultural University, Nanjing 210095, China; jiayuefang_zj@163.com (J.F.); 2021208003@stu.njau.edu.cn (W.D.); 2022108036@stu.njau.edu.cn (J.Z.); 9221810531@stu.njau.edu.cn (B.H.); 9221810517@stu.njau.edu.cn (Y.Z.); 19114243198@163.com (J.W.)

**Keywords:** *Lycium barbarum* L., 2-*O*-β-D-glucopyranosyl ascorbic acid, longevity regulation, DAF-16/HSF-1/SIR-2.1 pathway, transcriptomic, intestinal microbiota, *Caenorhabditis elegans*

## Abstract

2-*O*-β-D-Glucopyranosyl ascorbic acid (AA-2βG), a bioactive ascorbic acid derivative isolated from the fruits of *Lycium barbarum* L., exhibited significant antiaging effects in *Caenorhabditis elegans*. It significantly extended their lifespan, enhanced stress resistance, reduced lipofuscin accumulation, and improved their healthspan, while strengthening antioxidant defenses. Transcriptomic analysis identified the insulin/insulin-like growth factor (IGF)-1 signaling pathway as a key regulator, with quantitative real-time polymerase chain reaction confirming the upregulation of longevity-associated genes. Functional studies showed that the transcription factors DAF-16, HSF-1, and SIR-2.1 were essential for the lifespan-extending effects of AA-2βG, as mutations in these genes abolished lifespan extension. Moreover, 16S rRNA sequencing revealed that AA-2βG modulated gut microbiota by increasing longevity-associated taxa and reducing pro-aging species, with these alterations linked to metabolic pathways. These findings suggest that AA-2βG exerts antiaging effects through the coordinated regulation of the IIS pathway and gut microbiota composition, highlighting its potential as a natural geroprotective compound.

## 1. Introduction

Aging represents a systemic physiological decline characterized by oxidative damage, mitochondrial dysfunction, immune aging, and altered nutrient-sensing pathways [1]. As the global population ages, identifying effective antiaging strategies has become a central focus in biomedical research. While various synthetic chemical drugs are available, their long-term use may lead to potential side effects [2]. Recent research has highlighted the potential of natural plant compounds to slow the aging process, particularly those with potent antioxidant properties [3].

*Lycium barbarum* L., a traditional Chinese plant with both medicinal and dietary values, contains a diverse array of bioactive compounds, such as flavonoids, betaine, polysaccharides, and phenolic acids [4]. These compounds contribute to the diverse pharmacological characteristics, including antioxidant, anticancer, antiaging, antitumor, and immunomodulatory properties [5,6]. Among these bioactive compounds, 2-*O*-β-D-glucopyranosyl ascorbic acid (AA-2βG), a naturally occurring and derivative of ascorbic acid, has been identified in the fruits of *L. barbarum* [7]. The molecular structure of AA-2βG features a glucose moiety attached to the C2 hydroxyl group of the vitamin C (V_C_) molecule, which stabilizes the enediol structure responsible for oxidative susceptibility, preventing oxidation into dehydroascorbic acid. This modification enhances its stability while maintaining its function as a precursor to V_C_ [8]. Studies have demonstrated that AA-2βG possesses effective free radical scavenging properties, particularly against 2,2-diphenyl-1-picrylhydrazyl (DPPH) free radicals and hydroxyl peroxide [9], thus mitigating the cellular damage induced by oxidative stress. Given the well-established link between antioxidant activity and aging processes, AA-2βG shows promise as an antiaging agent, although its exact mechanisms need further investigation.

*Caenorhabditis elegans* has emerged as a classic model in aging research due to its short growth period, fully sequenced genome, and genetic tractability [10]. The insulin/insulin-like growth factor (IGF)-1 signaling (IIS) pathway is a key regulator of *C. elegans* lifespan, primarily through its modulation of the FOXO family homolog transcription factor DAF-16 [11]. Upon attenuation of IIS activity, DAF-16 is translocated to the nucleus, where it activates downstream genes responsible for oxidative stress resistance, heat shock responses, and metabolic homeostasis [12]. Longevity modulation in *C. elegans* also involves cross-talk between IIS and additional stress-responsive pathways [13]. For example, SIR-2.1, an NAD^+^-dependent sirtuin family histone deacetylase, serves as a FOXO/DAF-16-mediated transcriptional regulator under oxidative stress conditions [14]. Additionally, lifespan extension is often associated with enhanced resistance to various stressors; the transcription factor HSF-1, whose activation state is controlled by the IIS pathway, governs proteostasis maintenance and organismal longevity in *C. elegans* [15].

The gut microbiota, with core phyla such as Firmicutes and Bacteroidetes, is essential in regulating human health and disease [16]. Emerging research suggests that age-related dysbiosis of the gut microbiota may contribute to various aging-related pathologies. Notably, dietary interventions that modify gut microbial composition have been identified as key modulators of age-related health decline [17]. In this context, recent studies highlight the therapeutic potential of AA-2βG. It has been shown to significantly alleviate dextran sulfate sodium-induced colitis in mice by suppressing pro-inflammatory cytokines and modulating gut microbiota [18]. Furthermore, its neuroprotective effects against neuroinflammation induced by a high-fructose diet in mice models have been demonstrated [19]. These results suggest that AA-2βG’s antiaging mechanisms may involve microbiome-mediated pathways, potentially enhancing metabolic homeostasis, reducing systemic inflammation, and improving immune resilience, thereby promoting healthy aging. This study, therefore, aimed to systematically investigate the antiaging properties (effects on lifespan, stress resistance, gene expression patterns, and gut microbiota composition) and potential mechanisms of AA-2βG extracted from the fruits of *L. barbarum* in *C. elegans*. The results are expected to provide scientific evidence supporting the development of AA-2βG as a potential antiaging functional food ingredient.

## 2. Materials and Methods

### 2.1. Materials

The fruits of *L. barbarum* (variety, Ningnonggouqi No. 7) were kindly provided by the National Wolfberry Engineering Research Center (Yinchuan, China). Kits for determining superoxide dismutase (SOD), catalase (CAT), malondialdehyde (MDA), glutathione peroxidase (GSH-Px), and glutathione (GSH) were purchased from Nanjing Jiancheng Co., Ltd. (Nanjing, China). The Tissue Total RNA Isolation Kit and SYBR Green Kit were obtained from Nanjing Yifeixue Biotech Co., Ltd. (Nanjing, China). The nematode reproduction inhibitor 5-Fluoro-2′-deoxyuridine (FuDR) was purchased from Shanghai Sigma-Aldrich Co., Ltd. (Shanghai, China). Every other reagent was of analytical quality.

### 2.2. Preparation of AA-2βG

AA-2βG was prepared and characterized following our previously reported method [18]. The purity of the compound was assessed by high-performance liquid chromatography (HPLC) using an Agilent 1100 system (Agilent Technologies, Santa Clara, CA, USA) equipped with a diode array detector (DAD). The mobile phase consisted of 20% methanol, 1.2 mM phosphoric acid, and 5 mM tetrabutylammonium bromide, at a flow rate of 0.5 mL/min. The injection volume was 20 μL, and the total elution time was 15 min. Structural confirmation was performed by matrix-assisted laser desorption/ionization time-of-flight mass spectrometry (MALDI-TOF MS) (Applied Biosystems, Foster City, CA, USA) with 2,5-dihydroxybenzoic acid as the matrix.

### 2.3. Nematode Strains

N2 (wild type), CF1553 strain *muIs84* [(pAD76) *sod-3p*::GFP + *rol-6*(su1006)], TJ356 strain *daf-16*::GFP (zls356) IV, CF1038 strain *daf-16* (mu86) I*sir-2.1* (ok434) IV, *hsf-1* (sy441) I, and *Escherichia coli* OP50 were kindly provided by the College of Food Science and Technology, Nanjing Agricultural University, China. The nematodes were cultivated on nematode growth medium (NGM) agar plates according to normal techniques [20], with *E. coli* OP50 provided as a food source, and were maintained at 20 °C for 72 h to attain the L4 stage. The *E. coli* OP50 solutions containing different concentrations of V_C_ and AA-2βG were prepared. The nematodes were divided into the control group, the positive control group (V_C_, 5 mM), the AA-2βG low-dose group (AA-L, 2.5 mM), the AA-2βG medium-dose group (AA-M, 5 mM), and the AA-2βG high-dose group (AA-H, 7.5 mM).

### 2.4. Lifespan Analysis

Synchronized L4 nematodes were transferred to petri plates of the control, V_C_, AA-L, AA-M, and AA-H groups. To prevent interference from egg and larval development, the nematodes were transferred to fresh plates daily. Mortality was recorded every 24 h for each group until no nematodes remained alive. Death was confirmed when the nematodes were immobile, did not respond to a light touch with a picking needle, or failed to swallow. Nematodes that disappeared or died before reaching maturity were excluded from the analysis [20].

### 2.5. C. elegans Fertility Assay

Prior to egg-laying, synchronized nematodes were moved to brand-new NGM plates without FUdR, with a single worm on each plate. Throughout the spawning period, the nematodes were transferred to new culture dishes daily, while the original plates were retained to quantify the number of offspring hatched from the eggs. The duration of the egg-laying cycle generally spanned 5 to 7 days, and the total offspring produced was counted [21].

### 2.6. Assay of Lifespan Under Various Stressors

The stress resistance of the worms was evaluated using a modified protocol based on the method described by Zeng et al. [22]. For the heat stress experiments, the nematodes were placed in a 35 °C incubator, and mortality was recorded hourly. In the oxidative stress experiments, the nematodes were transferred to the plates with 0.1% of 30% H_2_O_2_, and survival rates were assessed every hour after exposure. In the ultraviolet (UV) stress experiments, the nematodes were exposed to UV irradiation (120 mJ/cm^2^) with survival rates monitored until all had perished. Mortality was determined following the protocols outlined in Section 2.4.

### 2.7. Measurement of Lipofuscin and Body Size in C. elegans

*C. elegans* at the L4 stage were cultured for 5 days prior to subsequent experiments. The nematodes from each group were randomly selected and imaged using a fluorescence microscope (Ex: 380 nm; Em: 430 nm, Leica Microsystems, Wetzlar, Germany) [23]. Lipofuscin fluorescence intensity and body length were measured by using ImageJ software (version 1.53t).

### 2.8. Body Bending and Pharyngeal Pumping Assays

Following the grouping and culturing protocols outlined in Section 2.4, the body bending frequency and the pharyngeal pumping rate of the nematodes were observed and counted for 20 s on day 5, 10, and 15 [23].

### 2.9. Motility Measurement

During the experiment, the locomotor abilities of the nematodes were evaluated on day 1, 10, and 15, and they were classified into three categories. Nematodes displaying spontaneous sinusoidal movement were assigned to category A, while those unable to perform sinusoidal movement but still capable of motion were classified as category B. Nematodes that only moved in response to external stimulation, with movement limited to the head and tail, were assigned to category C [24].

### 2.10. Reactive Oxygen Species (ROS) Assay in C. elegans

According to a previous study [25], synchronized L4 nematodes were cultured for 5 days and then incubated with dichlorodihydrofluorescein diacetate (DCFH-DA, 10 mM) in the dark at 20 °C for 30 min. After incubation, the nematodes were washed three times with M9 solution. Nematodes from each group were randomly imaged using a fluorescence microscope (Ex: 485 nm; Em: 438 nm).

### 2.11. Measurement of Biochemical Indexes in C. elegans

According to a previous study [25], synchronized nematodes cultured for 5 days were collected using M9 buffer, disrupted by ultrasonic treatment, and centrifuged at 3000 rpm for 10 min, affording the supernatants for analysis. Protein concentration was measured using a BCA protein assay kit. The SOD, CAT, and GSH-Px activities, and the levels of GSH and MDA in *C. elegans* were measured following the protocols provided in the respective assay kits.

### 2.12. mRNA Relative Expression Analysis

#### 2.12.1. Transcriptome Analysis

The transcriptome of control nematodes and nematodes treated with 7.5 mM AA-2βG for 5 days was analyzed. Approximately 2000 nematodes per experimental cohort were harvested from NGM agar plates and aggregated for subsequent processing. The RNA-seq library was constructed with three biological replicates. RNA extraction and transcriptome profiling were implemented by Personal Biotechnology Co., Ltd. (Shanghai, China). Total RNA was evaluated for purity and integrity prior to library construction using the NEBNext Ultra II RNA Library Prep Kit. Poly(A)+ mRNA was enriched, fragmented, and reverse-transcribed into cDNA, followed by the purification of 400–500 bp fragments. After quality control and quantification, the libraries were sequenced on an Illumina PE150 platform. Data processing involved quality filtering, genome alignment, and expression analysis. Differentially expressed genes were identified based on |log2FC| > 1 and *p* < 0.05.

#### 2.12.2. RNA Extraction and RT-qPCR Analysis

To validate key RNA expression changes identified in the transcriptome analysis, quantitative real-time polymerase chain reaction (RT-qPCR) was conducted. The synchronized L4 nematodes were collected after the treatment with 7.5 mM AA-2βG for 5 days, and total RNA was extracted using a silica column-based RNA extraction kit. Complementary DNA (cDNA) was synthesized from purified RNA, and RT-qPCR was conducted with a SYBR Green master mix. Gene expression levels were normalized to the reference gene glyceraldehyde-3-phosphate dehydrogenase (GAPDH) and quantified using the 2^−ΔΔCt^ method. Primers are listed in Table S1.

### 2.13. DAF-16::GFP Intracellular Localization

The relocation of DAF-16 to the nucleus is crucial for extending lifespan. To evaluate this process, the transgenic strain TJ356 was employed, where the DAF-16 protein was tagged with green fluorescent protein (GFP) to facilitate localization analysis. Referring to the method of Zhang et al. [23], nematodes from each group were randomly imaged using a fluorescence inverted microscope. Three localization patterns were defined: “Cytoplasmic” (no nuclear fluorescence), “Intermediate” (fluorescence concentrated in the head and tail regions), and “Nuclear” (punctate fluorescence throughout the body). The percentages of worms exhibiting each pattern were statistically compared between the treatment and control groups.

### 2.14. Gut Microbiota Analysis

According to Sun et al. [25], the gut microbiota of control nematodes and nematodes treated with 7.5 mM AA-2βG for 5 days were analyzed. Nematodes were subsequently washed, collected and stored at −80 °C, and four parallel samples were obtained per group. The bacterial 16S rRNA genes V3–V4 region was amplified via PCR using primers 338F (5′-ACTCCTACGGGAGGCAGCA-3′) and 806R (5′-GGACTACHVGGGTWTCTAAT-3′). Microbiome analysis was conducted using QIIME2 2022.11, encompassing sequence processing, ASV taxonomic classification, abundance filtering, and data visualization. Further analyses included alpha and beta diversity, taxonomic composition, functional potential prediction, and other related analyses.

### 2.15. Statistical Analysis

All the tests were repeatedly performed at least three times, and the results are expressed as the mean ± standard error of the mean (mean ± SEM). Differences between the two groups were analyzed using a two-tailed Student’s *t*-test, while one-way analysis of variance (ANOVA) followed by Tukey’s post hoc test was applied for multiple-group comparisons. A significance level of *p* < 0.05 was considered statistically significant.

## 3. Results and Discussion

### 3.1. AA-2βG Extended Lifespan of C. elegans

**Figure 1:**
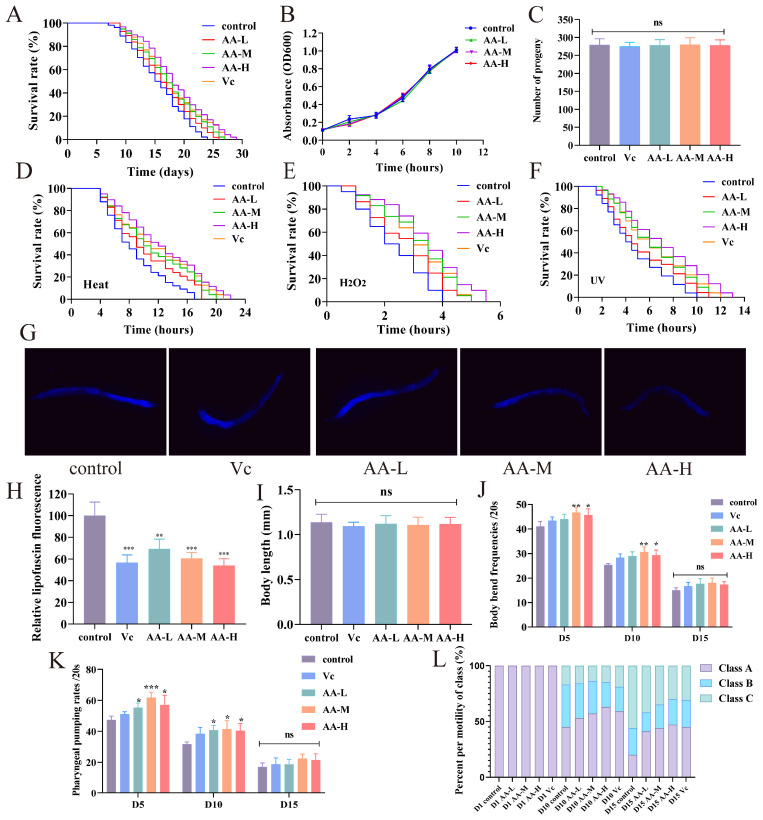
Effects of AA-2βG on *C. elegans* health indicators. (**A**) Survival curves of N2 treated with AA-2βG under normal growth conditions. (**B**) Growth dynamics of *E. coli* OP50 in LB medium containing 0, 2.5, 5.0, and 7.5 mM AA-2βG. (**C**) Reproductive outcomes of nematodes with and without AA-2βG treatment. (**D**–**F**) Effect of AA-2βG on nematode lifespan under heat, H_2_O_2_, and ultraviolet stress conditions. (**G**,**H**) Lipofuscin accumulation in *C. elegans* treated with different concentrations of AA-2βG. (**I**) Changes in body length of *C. elegans* after treatment. (**J**,**K**) Impact of AA-2βG on pharyngeal pumping and body bend frequency of *C. elegans*. (**L**) Changes in body size of AA-2βG-treated nematodes. * *p* < 0.05, ** *p* < 0.01, and *** *p* < 0.001, compared to the control.

Lifespan assessment provides a standardized method for evaluating the antiaging effects of bioactive substances [26]. Therefore, we used the lifespan analyses in *C. elegans* to evaluate the antiaging effects of AA-2βG. The results indicated that nematodes treated with AA-L, AA-M, and AA-H exhibited a longer average lifespan compared with untreated control (Figure 1A). Control group worms exhibited a median lifespan of 15.4 ± 0.2 days and a maximum lifespan of 23.4 ± 0.2 days. After treatment with AA-L, AA-M, and AA-H, the median lifespan increased to 16.1 ± 0.3 days (maximum 25.2 ± 0.4 days), 17.3 ± 0.2 days (maximum 26.9 ± 0.4 days), and 18.4 ± 0.2 days (maximum 28.9 ± 0.4 days), respectively (Table S2). The protection level of the AA-M was comparable to that of V_C_, while the AA-H elevated median longevity by 5.7% compared with V_C_. These findings suggest that AA-2βG promoted significant lifespan extension in *C. elegans* at both medium and high concentrations, providing preliminary evidence of the antiaging effects of AA-2βG.

As previously reported, the pursuit of longevity in living organisms is often accompanied by trade-offs such as reduced somatic maintenance, caloric restriction, or impaired fertility [27]. The bacterial proliferation assays showed that bacterial growth was not inhibited by AA-2βG (Figure 1B), indicating that the antiaging mechanism was not mediated by suppressed food consumption. To further assess the potential influence of AA-2βG on nematode reproduction, larval offspring were quantified. No substantial variation was detected between the AA-2βG and control groups (Figure 1C), confirming that the lifespan extension induced by AA-2βG occurred independently of reproductive modulation.

These findings suggest that the lifespan-prolonging effects of AA-2βG in *C. elegans* are not attributable to conventional non-genetic factors but rather involve the direct modulation of intrinsic aging-related signaling pathways. Consistent with this, previous studies have shown that natural compounds such as the anthocyanin monomer petunidin-3-*O*-[rhamnopyranosyl-(trans-*p*-coumaroyl)]-5-*O*-(β-D-glucopyranoside) and Lonicera japonica polysaccharides can prolong the lifespan of *C. elegans* without compromising food intake or fertility [23,28].

### 3.2. AA-2βG Enhanced Stress Resistance in C. elegans

Previous study has shown that lifespan extension in *C. elegans* is closely linked to enhanced adaptive responses to external stressors [29]. This evidence suggests that AA-2βG may rejuvenate early-life resilience mechanisms to improve stress resistance. To test this hypothesis, *C. elegans* were subjected to extreme environmental challenges. In thermal stress assays (35 °C exposure), significant lifespan extension was observed in *C. elegans* treated with AA-2βG (Figure 1D). The median lifespan of *C. elegans* treated with AA-L, AA-M, and AA-H, as well as V_C_, was extended by 8.3%, 22.6%, 41.7%, and 35.7% (Table S2), respectively. Under acute oxidative stress, AA-L, AA-M, and AA-H treatment extended the median lifespan of the nematodes by 31.8%, 54.5%, and 63.6%, respectively (Figure 1E and Table S2). This dose-dependent response confirmed the capacity of AA-2βG to enhance thermotolerance in *C. elegans*. Under UV-B stress, the median lifespan of *C. elegans* treated by AA-M, AA-H, and V_C_, significantly increased by 45.2%, 71.4%, and 50.1%, respectively (Figure 1F and Table S2). Moreover, the lifespan extension effect of AA-H was comparable with that of V_C_, demonstrating that AA-2βG strengthened stress resistance and promoted lifespan in *C. elegans*.

The results showed that AA-2βG significantly enhanced the stress resistance of *C. elegans* under various environmental conditions, including heat, oxidative, and UV-B-induced stress conditions. Specifically, heat stress simulates elevated temperatures that induce protein denaturation and secondary ROS production [30], leading to the accumulation of intracellular ROS [31], whereas UV-B irradiation inflicts direct DNA damage and contributes to photo-oxidative stress [32]. The ability of AA-2βG to confer protection across these diverse stress models implied that it enhanced antioxidative defense mechanisms and pathways involved in proteostasis maintenance and DNA repair, thereby collectively contributing to improved stress resilience and delayed aging in *C. elegans*.

### 3.3. AA-2βG Reduced Lipofuscin and Showed No Effect on Body Size in C. elegans

Auto-fluorescence analysis revealed a significant attenuation of lipofuscin deposition in AA-2βG-treated nematodes compared with control nematodes. All the AA-2βG-treated nematodes exhibited a statistically significant reduction in lipofuscin level, with the AA-H group displaying the most substantial reduction (Figure 1G,H). These findings established AA-2βG as a potent suppressor of age-related lipofuscinogenesis, with efficacy surpassing canonical antioxidants at higher concentrations. Regarding the body size of *C. elegans*, no substantial differences were found between the control and AA-2βG groups (Figure 1I). Lipofuscin, an autofluorescent aggregate composed of oxidized proteins, lipids, and metal ions, progressively accumulates during aging as a result of impaired proteolytic systems and oxidative stress [23]. Since lipofuscin accumulation is a well-established marker of cellular aging, the observed reduction in lipofuscin levels following AA-2βG treatment suggested that AA-2βG might alleviate stress-induced cellular damage, thereby contributing to the delay of aging processes in *C. elegans*.

### 3.4. AA-2βG Improved the Health Span of C. elegans

It has been reported that the motility of nematodes is a direct indicator of their health status, as enhanced movement facilitates an optimal feeding rate [33]. To evaluate AA-2βG-mediated health span improvements, we quantified two critical behavioral metrics: body bending and pharyngeal pumping rate of *C. elegans.* As shown in Figure 1J,K, AA-2βG treatment significantly increased both metrics compared with the control group on day 5 and day 10, corresponding to the early and mid-survival phases of nematodes. Notably, the AA-M group showed the most pronounced effect. Furthermore, we also evaluated the motility of nematodes based on muscle-driven movement capacity. The results revealed that on day 15, more than 40% of the nematodes treated with AA-2βG and V_C_ were classified as group A, while only about 20% of the control group were classified as group A.

A central objective of antiaging research is to extend the healthy phase of life by preserving functional abilities and limiting cellular degeneration [33]. The observed improvements in locomotion metrics suggested that AA-2βG might counteract the age-associated decline in motor function, possibly through mechanisms related to muscle maintenance or neuromuscular signaling, thereby contributing to an enhanced health span in *C. elegans.*

### 3.5. AA-2βG Enhanced Antioxidant Capacity in C. elegans

**Figure 2:**
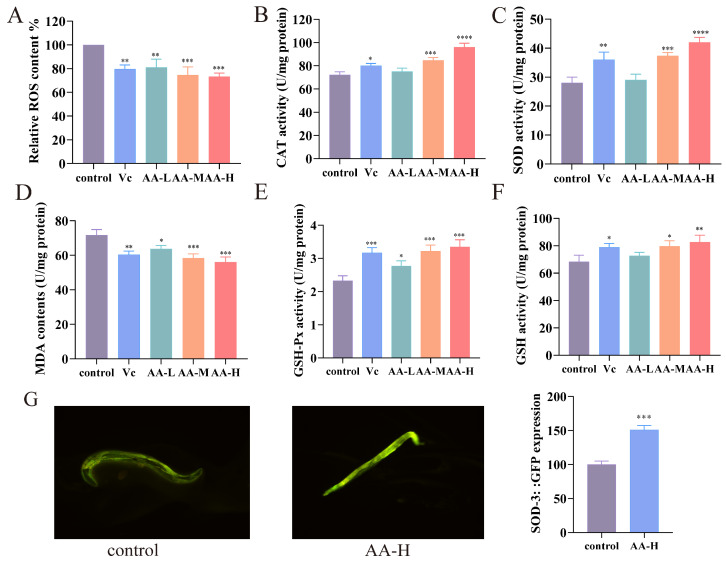
AA-2βG enhanced antioxidant capacity in *C. elegans.* (**A**) Fluorescence quantification of ROS in *C. elegans*. (**B**–**F**) Effects of AA-2βG on the activities of CAT, SOD, MDA, GSH-Px and GSH content in *C. elegans*. (**G**) Fluorescence images and relative fluorescence intensity in CF1553 (SOD-3::GFP). * *p* < 0.05, ** *p* < 0.01, *** *p* < 0.001, and **** *p* < 0.0001, compared to the control.

**Figure 3:**
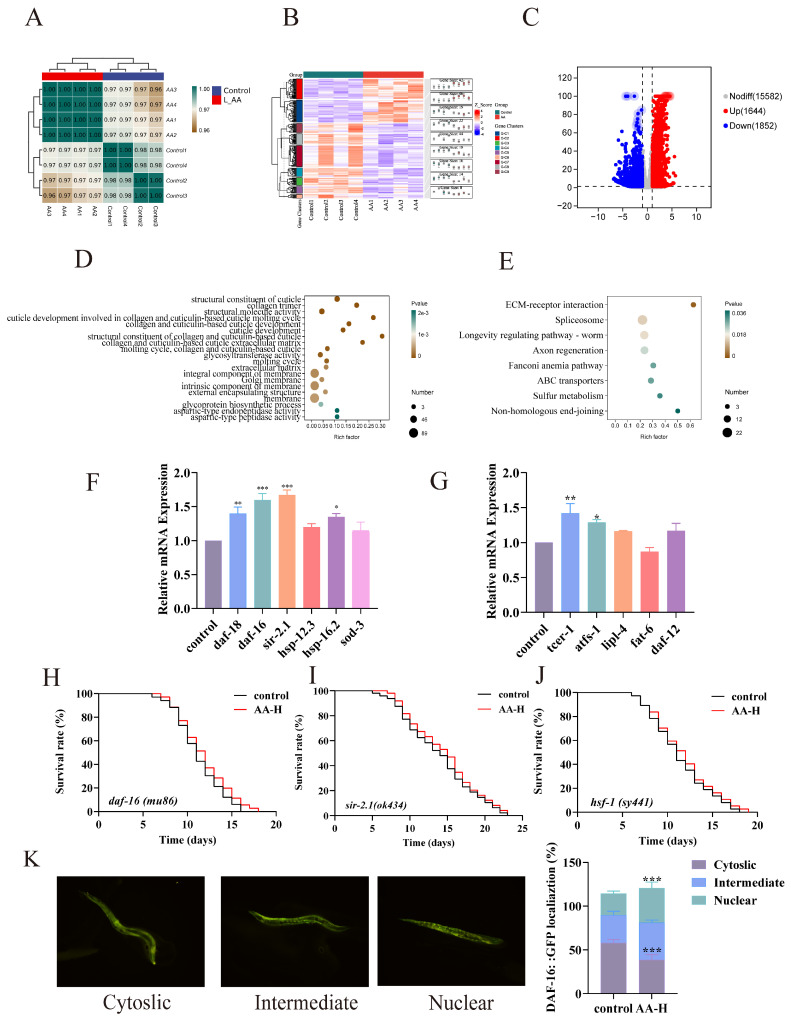
Effects of AA-2βG on the transcriptome of *C. elegans*. (**A**) Pearson correlation coefficients analysis between different biological repetitions. (**B**) Volcano plot of differentially expressed genes between different groups; the most significant changes in gene supplementation are presented in Table S3. (**C**) Cluster analysis of DEGs among control and AA-2βG groups. (**D**) KEGG enrichment analysis of different groups. (**E**) GO enrichment analysis of different groups. (**F**,**G**) Effect of AA-2βG on the mRNA expressions of aging-related genes. (**H**) Representative fluorescence images and quantification of DAF-16GFP localization. (**I**–**K**) Lifespan of mutant nematodes *mu86 (daf-16)*, *ok434 (sir-2.1)*, and *sy441 (hsf-1)* treated with AA-2βG. * *p* < 0.05, ** *p* < 0.01, and *** *p* < 0.0001, compared to the control.

AA-2βG treatment significantly reduced ROS accumulation in *C. elegans* in a dose-dependent manner (*p* < 0.05) (Figure 2A). Concurrently, the values of CAT and SOD were markedly elevated in nematodes treated with AA-M and AA-H relative to the control group, indicating an enhanced endogenous antioxidant defense system (Figure 2B,C). Furthermore, MDA levels in the AA-M and AA-H groups were significantly lower than those in the control group (Figure 2D), suggesting a substantial reduction in oxidative damage. In parallel, elevated levels of reduced GSH and increased GSH-Px activity were observed, demonstrating a coordinated augmentation of both enzymatic and non-enzymatic antioxidant pathways (Figure 2E,F). These results revealed that AA-2βG effectively enhanced the antioxidant activity of enzymatic and non-enzymatic substances in nematodes. Remarkably, fluorescence quantification in CF1553 (SOD-3::GFP) nematodes revealed that AA-2βG supplementation induced a 51.23% intensity increase (Figure 2G), and an upregulation in SOD-3 expression was observed (Figure 3F), substantiating the improved antioxidant capacity of the nematodes.

Oxidative stress is a major contributor to aging and related diseases, primarily due to excessive ROS that overwhelms intrinsic antioxidant defenses [31]. In *C. elegans*, the antioxidant defense system consists of enzymatic factors (SOD, CAT, and GSH-Px) and the non-enzymatic molecule GSH, which collectively maintains redox homeostasis and delays senescence. Considerable research has explored the ability of plant-derived compounds to prolong nematode lifespan via their antioxidant effects [34]. Our findings demonstrated that AA-2βG significantly reduced oxidative stress markers and concurrently enhanced both enzymatic and non-enzymatic antioxidant defenses in *C. elegans*, suggesting its potential to alleviate ROS-induced cellular damage and support lifespan extension.

Structurally, AA-2βG is a β-glycosylated derivative of ascorbic acid. This modification enhances chemical stability and prolongs biological activity under physiological conditions [8]. Compared to V_C_, which acts as a fast-acting antioxidant, AA-2βG is more inclined to function as a slow-acting antioxidant [35]. This sustained redox modulation capability may provide AA-2βG with a unique pharmacological advantage in antiaging applications, enabling the continuous regulation of redox signaling and stress response pathways that are critical for aging control.

### 3.6. AA-2βG Modulated the Expression of Related mRNA in C. elegans

To further investigate the mechanism underlying AA-2βG’s capacity to extend the longevity of *C. elegans*, transcriptomic analysis was performed. High correlation among the samples (Pearson correlation coefficient > 0.98) confirmed the consistency of the experiment (Figure 3A). Subsequently, cluster analysis was conducted to evaluate the overall expression profiles of differentially expressed genes (DEGs) across the groups. The heatmap revealed notable alterations in gene expression between the AA-2βG-treated and control groups (Figure 3B). Differential gene expression analysis revealed that AA-2βG treatment induced 3496 differentially expressed genes relative to the control, including 1644 upregulated and 1852 downregulated genes (Figure 3C).

Gene ontology (GO) enrichment analysis was used to interpret the results across three categories: molecular function (MF), cellular component (CC), and biological process (BP) (Figure 3D). The results revealed that in the MF category, upregulated genes were mainly enriched in the structural constituent of cuticles. In the CC category, upregulated genes were mainly enriched in the collagen trimer and extracellular matrix. In the BP category, upregulated genes were primarily enriched in categories related to the cuticle molting cycle, glycoprotein biosynthesis regulation, and collagen metabolism. These diverse biochemical processes and signaling pathways are closely related to tissue integrity maintenance, metabolic homeostasis regulation, and cell damage repair during aging [36], further supporting the hypothesis that AA-2βG delayed aging through multifaceted mechanisms.

Subsequently, Kyoto Encyclopedia of Genes and Genomes (KEGG) pathway analysis was conducted further to characterize the functional features of expressed genes. The results demonstrated that in the genetic information processing category, the longevity-regulating pathway in *C. elegans* was significantly enriched (Figure 3E), providing further evidence of the lifespan-extending effects of AA-2βG. Regarding environmental information processing, the pronounced activation of the extracellular matrix (ECM)–receptor interaction pathway was in line with GO analysis, suggesting that AA-2βG might maintain tissue homeostasis by modulating the cellular microenvironment [37]. The coordinated activation of the Spliceosome, Fanconi anemia pathway, and non-homologous end-joining pathway in the genetic information processing category indicated AA-2βG’s potential to maintain genomic stability and neuronal maintenance [38]. Moreover, in metabolic regulation, enrichment of the sulfur metabolism pathway indicated that AA-2βG optimized detoxification processes, contributing to metabolic homeostasis during aging [39]. These coordinated actions across multiple pathways suggested that AA-2βG exerted its antiaging effects by targeting longevity regulation, cellular repair, and metabolic regulation pathways.

### 3.7. Genetic Requirements for AA-2βG-Mediated Antiaging Effects

Transcriptomic analysis revealed that AA-2βG modulated multiple signaling pathways related to lifespan regulation, stress resistance, and metabolic homeostasis. To validate these findings, we performed RT-qPCR analysis to examine the expression levels of representative genes. Notably, AA-2βG treatment significantly upregulated *daf-16* and *daf-18*, which are the central components of the IIS pathway (Figure 3F). DAF-16, a FOXO family transcription factor, plays a pivotal role in delaying aging by activating a suite of genes related to oxidative stress defense and longevity [40] and may serve as a central mediator of AA-2βG’s effects in *C. elegans*. Under normal physiological conditions, DAF-16 is phosphorylated and sequestered in the cytoplasm. However, when IIS is reduced, it translocates to the nucleus to promote the transcription of protective genes [41]. The results from the RT-qPCR analysis showed that the expression of *sod-3*, *sir-2.1*, and *smk-1*, which are the downstream antioxidant genes of *daf-16*, was significantly increased. SIR-2.1, an NAD^+^-dependent histone deacetylase and a cofactor of DAF-16/FOXO signaling, may affect the lifespan of *C. elegans* through its role in stress response modulation [42]. HSF-1, a master transcriptional effector of the IIS pathway, orchestrates heat shock responses by directly activating molecular chaperones. AA-2βG was also found to upregulate *hsp-16.2* and *hsp-12.3* (Figure 3F), which are directly regulated by HSF-1, suggesting the potential regulatory involvement of HSF-1 in mediating these effects [43]. The activation of these downstream resistance factors and antioxidant-related genes further explained the enhanced antioxidant capacity and improved tolerance to extreme conditions in nematodes, thereby contributing to their extended lifespan.

The genes related to fatty acid metabolism, such as *tcer-1*, *lipl-4*, and *daf-12*, were upregulated by AA-2βG, while *fat-6* was downregulated (Figure 3G). These results suggested that AA-2βG might help maintain cellular metabolic homeostasis and thereby contributed to lifespan extension. Among these metabolic regulators, the nuclear hormone receptor DAF-12 plays a crucial role in the integration of reproductive and metabolic signals to control the lifespan and dauer formation through lipid metabolic reprogramming [44]. The lysosomal acid lipases TCER-1 and LIPL-4 can regulate lipid droplet dynamics, promote lipolysis and lipid recycling, and activate autophagy-related genes, thereby specifically extending the lifespan of nematodes [45,46]. These changes in the expression of metabolism-related genes induced by AA-2βG likely reduced age-associated lipid accumulation, thereby attenuating lipotoxicity and extending the lifespan. In addition, the *atfs-1* was upregulated by AA-2βG (Figure 3G), suggesting that AA-2βG activates the mitochondrial unfolded protein response (UPRmt) pathway via ATFS-1, a master regulator of mitochondrial proteostasis [47]. Its upregulation may repair damaged mitochondria and enhance their function, thereby mitigating age-related mitochondrial decline and extending the lifespan.

### 3.8. Validation of Fluorescent Mutants

Notably, the upregulation of *daf-16* and *sir-2.1* was the most pronounced among the IIS-related genes, and the activation of *hsp-16.2* and *hsp-12.3* suggested that AA-2βG might extend lifespan by activating *hsf-1*. To validate the mechanism by which AA-2βG extended lifespan through specific gene regulation, mutant nematodes *mu86 (daf-16)*, *ok434 (sir-2.1)*, and *sy441 (hsf-1)* were treated with 7.5 mM AA-2βG to evaluate the roles of the three genes in longevity. In *daf-16* mutant nematodes, only a 2.9% lifespan extension was observed for the AA-2βG treatment, which exhibited no significant difference in lifespan compared with the control (Figure 3H), suggesting that DAF-16 was essential for the pro-longevity effect of AA-2βG. Moreover, lifespan assays using *sir-2.1* and *hsf-1* mutants revealed that the treatment with 7.5 mM AA-2βG extended the median lifespan of *C. elegans* by 4.3% and 5.6%, respectively (Figure 3I,J). Compared with N2 wild-type nematodes, in which lifespan was extended by over 20%, AA-2βG exhibited reduced lifespan extension effects in these mutants, indicating that the longevity-regulating effect of AA-2βG was partially dependent on SIR-2.1 and HSF-1. To further verify the functional status of the DAF-16 transcription factor, the *C. elegans* mutant strain *daf-16(mu86)* was used to determine whether AA-2βG promotes the nuclear translocation of DAF-16.The results revealed that AA-2βG treatment significantly promoted the nuclear translocation of DAF-16 protein (Figure 3K), a necessary step for the lifespan-extending effect, confirming the essential role of DAF-16 in mediating the stress resistance and lifespan extension effects induced by AA-2βG. In conclusion, AA-2βG exerted antiaging effects by promoting the nuclear accumulation and transcriptional activity of DAF-16, with SIR-2.1 and HSF-1 acting as critical co-factors in the IIS pathway. AA-2βG also regulated metabolism-associated genes to enhance the organism’s antioxidant defense system, stress tolerance, and metabolic homeostasis, ultimately delaying aging through multi-target synergistic mechanisms.

### 3.9. AA-2βG Regulated the Gut Microbiota of C. elegans

**Figure 4:**
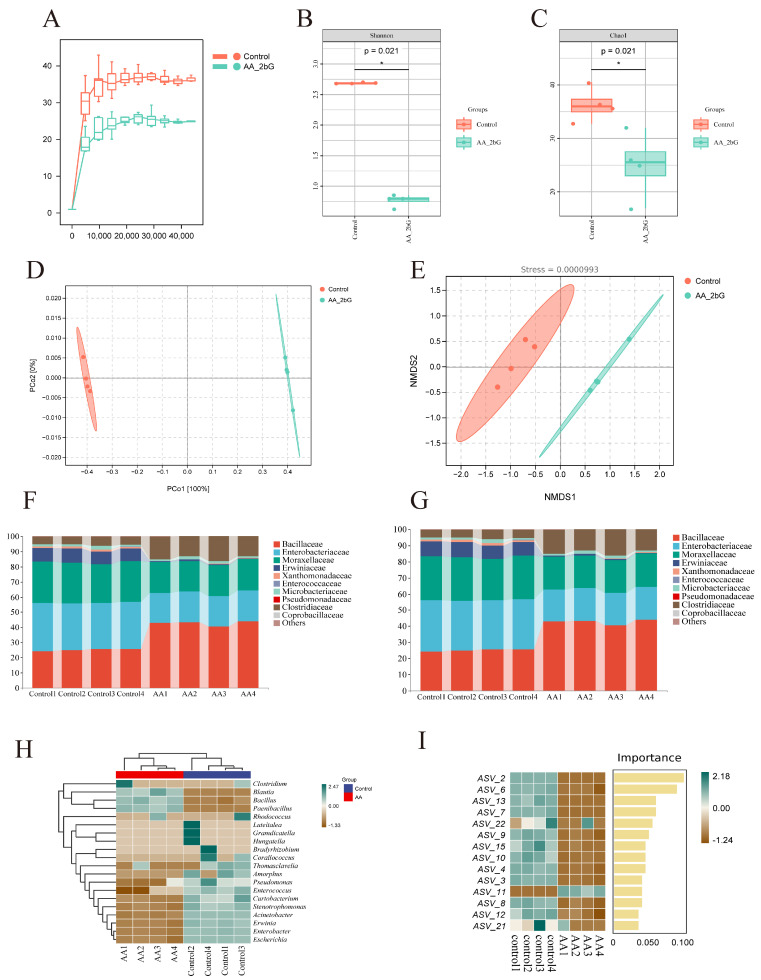
AA-2βG regulated the gut microbiota of *C. elegans*: (**A**) rarefaction curves; (**B**) Shannon index; (**C**) Chao1 index; (**D**,**E**) PCoA and NMDS of gut microbiota in the control and AA-2βG groups; (**F**,**G**) taxonomic profiling of intestinal bacteria at the phylum level and family level; (**H**) heatmap of the distribution trend of genus abundance; (**I**) indicator species based on ASV/OTU for inter-group differences and their importance in descending order.

Recent studies have underscored the essential role of gut microbiota in sustaining host health and regulating physiological functions. Dietary interventions targeting the gut microbiota have been shown to benefit host health [48]. To evaluate the impact of AA-2βG on gut microbiota, 16S rRNA gene sequencing was employed to analyze the composition and function of the nematode intestinal microbiome. The rarefaction curves indicated that the sequencing depth for both groups approached saturation (Figure 4A), suggesting high data reliability. To better understand the variations in microbial community diversity among the three groups, we employed the Shannon index (Figure 4B) and Chao1 (Figure 4C) index to evaluate the alpha diversity. The results revealed notable differences between the AA-2βG-treated and control groups. Despite the reduced alpha diversity, AA-2βG treatment likely promoted a shift toward a functionally beneficial microbial composition. Beta diversity analysis further supported differences between groups: both PCoA and NMDS analyses (stress value < 0.2) demonstrated distinct separation in the microbiota composition between the AA-2βG and control groups (Figure 4D,E).

Based on species annotation data, the top 10 species with the highest relative abundance at both the phylum and family levels were identified within each group, and a bar chart was then constructed to visualize their relative abundances. At the phylum level, the predominant bacterial phyla in the nematode gut were Proteobacteria, Firmicutes, Actinomycetota, and Bacteroidetes (Figure 4F), consistent with previous research on nematode gut microbiota composition [49]. The relative abundance of Firmicutes increased significantly, while the abundance of Proteobacteria decreased following AA-2βG treatment, suggesting that this intervention might optimize the gut microbiota structure, promoting a configuration more favorable for energy metabolism and host homeostasis. At the Family level, AA-2βG supplementation resulted in an increased prevalence of Bacillaceae and Clostridiaceae, while the relative proportions of Erwiniaceae, Moraxellaceae, and Enterobacteriaceae decreased (Figure 4G).

Additionally, the top 20 OTUs with the highest absolute abundance across all samples were selected for correlation analysis with genus-level annotation results. The dominant characteristic bacteria in the AA-2βG group included *Bacillus*, *Paenibacillus*, and *Blautia*. Species of *Bacillus* have been shown to enhance intestinal barrier integrity and suppress pathogen colonization through the secretion of antimicrobial peptides and short-chain fatty acids (SCFAs), highlighting their potential as probiotics [50]. Of particular significance, *B. subtilis* extends the longevity of *C. elegans* through stimulation of the DAF-2/DAF-16/HSF-1 signaling cascade and suppression of insulin-like signaling pathway activity [51]. Similarly, *Blautia*, a core butyrate-producing genus, regulates lipid metabolism through AMPK pathway activation, with its metabolic byproduct butyrate directly linked to improved intestinal barrier function and longevity [52]. Additionally, *Paenibacillus*, the preferred dietary bacterium for *C. elegans*, demonstrates marked anti-inflammatory effects, potent antioxidant activity, and immune-regulating capabilities [53]. In contrast, the control group was characterized by *Thomasclavelia*, *Amorphus*, *Pseudomonas*, *Erwinia*, *Enterococcus*, *Curtobacterium*, *Stenotrophomonas*, *Acinetobacter*, *Erwinia*, *Enterobacter*, and *Escherichia* (Figure 4H). These findings are consistent with previous reports, which identified *Pseudomonas* and *Stenotrophomonas* as the main gut microbiota in normal worms [49].

**Figure 5:**
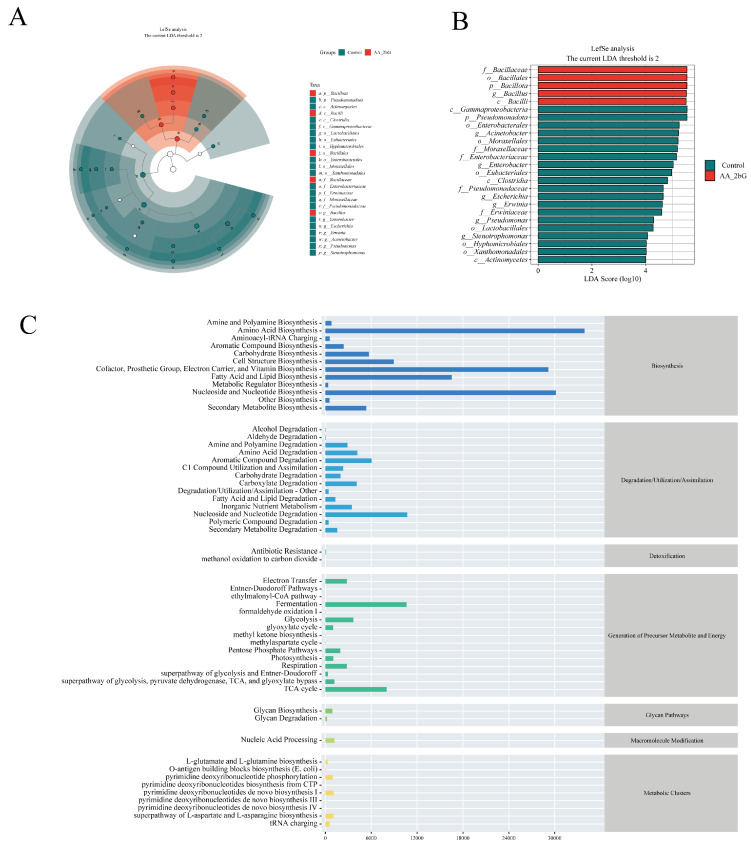
AA-2βG regulated the gut microbiota of *C. elegans*. (**A**,**B**) The LEfSe analysis of gut microbiota between the control and AA-2βG groups. (**C**) Abundance of metabolic pathways based on PICRUSt2 analysis.

Random forest analysis identified key regulatory effects of AA-2βG on the gut microbiota of *C. elegans*, 14 ASVs with significant abundance difference between groups were identified (Figure 4I and Table S4). To further analyze the variations in gut microbiota composition between the groups, a differential bacterial comparison analysis was conducted using an LDA score threshold of 4. As illustrated in Figure 5A,B, twenty-five taxa with significant abundance were identified. The relative abundance of *Bacillus* significantly increased in the AA-2βG group, aligning with the trends observed in the species composition analysis. Conversely, the relative abundances of *Acinetobacter*, *Enterobacter*, *Escherichia*, *Erwinia*, *Pseudomonas*, and *Stenotrophomona* decreased. Studies have shown that *Acinetobacter* accelerates aging in *C. elegans* through hyperactivation of Toll-like receptor-associated innate immune pathways [54], while *Enterococcus* compromises intestinal barrier integrity via secretion of MHC-I-targeting Epx pore-forming toxin probiotic colonization [55]. In particular, the *Pseudomonas aeruginosa* strain PA14, a pathogen previously demonstrated to infect mice and plants, has been reported to exhibit lethal effects in *C. elegans* [56]. Collectively, AA-2βG-mediated suppression of these microbes, coupled with the enrichment of beneficial microbes, restructured the gut microbiota to enhance metabolic efficiency, attenuate systemic inflammation, and synergistically extend longevity.

### 3.10. Functional Predictive Analysis

Based on 16S rRNA gene sequencing results, PICRUSt2 was employed to predict metabolic functional changes in the intestinal microbiota of *C. elegans*. Following AA-2βG intervention, significant enrichment was observed in eight metabolic pathways within the gut microbiota, including fatty acid and lipid biosynthesis and degradation, amino acid biosynthesis, nucleoside and nucleotide synthesis, and other pathways (Figure 5C). Gut microbiota-derived metabolites are widely acknowledged as essential for maintaining health and contributing to disease progression [57]. By reshaping the gut microbiota’s metabolic functions, AA-2βG promoted healthy aging in the host. Studies have demonstrated that lipid metabolism plays a pivotal role in the aging process [58]. Dietary regulation and other interventions targeting lipid metabolism have been shown to effectively extend the lifespan of various model organisms, including nematodes [59]. Specifically, AA-2βG enriched fatty acid and lipid biosynthesis and degradation pathways, suggesting that it regulated *C. elegans* lifespan by modulating lipid metabolic balance, which aligns with transcriptomic findings. Furthermore, the enrichment of amino acid biosynthesis pathways indicated that microbiota-derived amino acid metabolites might influence host health by regulating immunity, cellular functions, and microbial composition [60]. Additionally, the upregulation of nucleoside and nucleotide synthesis indicated that AA-2βG could support RNA synthesis and DNA replication, thereby promoting cellular growth and division and delaying aging processes [61]. The overall changes in these metabolic functions revealed the directional regulatory influence of AA-2βG on the metabolic network of gut microbiota.

These findings highlight the potential role of AA-2βG in modulating the gut microbiota’s metabolic functions, suggesting its capacity to maintain metabolic homeostasis and lifespan modulation.

## 4. Conclusions

The antiaging properties of AA-2βG were systematically evaluated in *C. elegans*, a widely utilized model for aging research. It was found that AA-2βG extended *C. elegans* lifespan and mitigated age-related functional decline. Additionally, AA-2βG treatment boosted antioxidant enzyme activity and enhanced stress resistance in nematodes. Transcriptomics results revealed that AA-2βG upregulated the expression of key genes (e.g., *daf-16*, *daf-18*, *sir-2.1*, *sod-3*, and *hsp-16.2*) in the IIS pathway and modulated metabolism-related genes. Notably, the essential roles of *daf-16*, *sir-2.1*, and *hsf-1* in lifespan extension within the IIS pathway were confirmed through validation experiments using relevant mutant strains. Further gut microbiota analysis showed that AA-2βG supplementation drove the enrichment of beneficial genera while reducing the abundance of potentially harmful genera, correlating with enhanced antioxidant capacity and metabolic homeostasis. This study provides new insights into the molecular mechanisms by which AA-2βG mediates lifespan extension, offering a compelling basis for the development of functional foods with potent antiaging properties. These findings lay the groundwork for further investigations into the therapeutic potential of AA-2βG in combating age-related diseases and promoting healthy aging.

## Data Availability

The original contributions presented in this study are included in the article/Supplementary Materials. Further inquiries can be directed to the corresponding author.

## Supplementary Materials

The following supporting information can be downloaded at https://www.mdpi.com/article/10.3390/foods14111875/s1: Table S1. Primer sequences used in RT-qPCR. Table S2. Effects of AA-2βG on the lifespan of *C. elegans* (mean ± SD, n = 3). Table S3 Supplementary chart for transcriptome volcano plot (3C), displaying the top 20 genes with the greatest significant differences (the top 20 discrete points in the volcano map). Table S4 Summary of specific species of 14 ASVs.
