# Supplementary material for: Antiaging Effect of 2-O-β-D-Glucopyranosyl Ascorbic Acid Derived from Lycium barbarum L. Through Modulating the IIS Pathway and Gut Microbiota in Caenorhabditis elegans
Source: Foods. 2025 May 25;14(11):1875. doi: 10.3390/foods14111875 (PMC12154002; doi:10.3390/foods14111875)
Supplement: Supplementary file 1 [file foods-14-01875-s001.zip › foods-3608888-supplementary.pdf]

Antiaging effect of 2-*O*-β-D-glucopyranosyl ascorbic acid derived from *Lycium barbarum* L. through modulating the IIS signaling pathway and gut microbiota in *Caenorhabditis elegans*

Jiayue Fang, Wei Dong, Jingqian Zheng, Baixuan Han, Yuying Zhang, Jianing Wang, Xiaoxiong Zeng [*]

College of Food Science and Technology, Nanjing Agricultural University, Nanjing 210095, Jiangsu, China

[*] Corresponding author: Tel/Fax: +86-25-84396791; E-mail: zengxx@njau.edu.cn (X. Zeng)

Table S1. Primer sequences used in RT-qPCR.

Genes	Forward primer (5’ to 3’)	Reverse primer (5’ to 3’)
act-1	GGAGTCATGGTCGGTATGG	CTTGAGGGTAAGGATACCTCTC
daf-18	CGCGAGGAGGAGAGGAGG	TGTGGAAGGAGAGGAGGA
daf-16	TCCATCATCTTTCCGTCCC	CTTCCAATAGCTGGAGAAACAC
sir-2.1	TACATTGCTCGAAGTGCCGA	TCAGATGGTAGCGGCGAATC
hsp-12.3	TTGTTAAGGTTCTGGACTACGA	TCACATCAATCTCGTTTGGC
hsp-16.2	TGCCATCAATCTCAACGTCTCACAG	CTTCGACGATTGCCTGTTGAATTGG
sod-3	ATGGACACTATTAAGCGCGA	GCCTTGAACCGCAATAGTG
tcer-1	TGTGGAAGGAGAGGAGGAG	CGTGAAGGAGGAAGGAGGA
daf-12	AGGCGTTTCGTCAAAGTTGC	CCTGCTCTCCGAACAACGAT
atfs-1	ATGGTGAGCAAGGGCGAGGAG	TTACTTGTACAGCTCGTCCAT
lipl-4	ATGGTGAGCAAGGGCGAGGAG	TTACTTGTACAGCTCGTCCAT
fat-6	ATGGTGAGCAAGGGCGAGGAG	TTACTTGTACAGCTCGTCCAT

Table S2. Effects of AA-2βG on the lifespan of *C. elegans* (mean ± SD, n = 3)

No.	Group	Median lifespan	Maximum lifespan	Compared with No.	p (log-rank test)
1	Control (Normal)	15.4 ± 0.2 (d)	23.4 ± 0.2 (d)	/	/
2	AA-L (Normal)	16.1 ± 0.3 (d)	25.2 ± 0.4 (d)	1	0.1684
3	AA-M (Normal)	17.3 ± 0.2 (d)	26.9 ± 0.4 (d)	1	0.0248
4	AA-H (Normal)	18.4 ± 0.2 (d)	28.9 ± 0.4 (d)	1	0.001
5	V_C (Normal)	17.4 ± 0.3 (d)	27.2 ± 0.3 (d)	1	0.0135
6	Control (Heat)	8.4 ± 0.5 (h)	17.1 ± 0.2 (h)	/	/
7	AA-L (Heat)	9.1 ± 0.3 (h)	17.8 ± 0.2 (h)	5	0.2523
8	AA-M (Heat)	10.3 ± 0.2 (h)	20.4 ± 0.3 (h)	5	0.0801
9	AA-H (Heat)	11.9 ± 0.3 (h)	22.2 ± 0.3 (h)	5	0.0048
10	V_C (Heat)	11.4 ± 0.2 (h)	21.3 ± 0.4 (h)	5	0.0421
11	Control (H_2O_2)	2.2 ± 0.2 (h)	4.1 ± 0.2 (h)	/	/
12	AA-L (H_2O_2)	2.9 ± 0.5 (h)	4.4 ± 0.2 (h)	11	0.2609
13	AA-M (H_2O_2)	3.4 ± 0.2 (h)	5.0 ± 0.3 (h)	11	0.0252
14	AA-H (H_2O_2)	3.6 ± 0.2 (h)	5.6 ± 0.3 (h)	11	0.0037
15	V_C (H_2O_2)	3.1 ± 0.2 (h)	5.1 ± 0.4 (h)	11	0.0342
16	Control (UV)	4.2 ± 0.2 (h)	4.1 ± 0.2 (h)	/	/
17	AA-L (UV)	4.7 ± 0.3 (h)	4.4 ± 0.2 (h)	16	0.4554
18	AA-M (UV)	6.1 ± 0.2 (h)	5.0 ± 0.3 (h)	16	0.049
19	AA-H (UV)	7.2 ± 0.2 (h)	5.6 ± 0.3 (h)	16	0.0052
20	V_C (UV)	6.3 ± 0.2 (h)	5.1 ± 0.4 (h)	16	0.0425

Table S3. Supplementary chart for transcriptome volcano plot (3C), displaying the top 20 genes with the greatest significant differences (the top 20 discrete points in the volcano map).

WBGene id	Gene name	Base mean	foldChange (AA-2βG/Control)	pval	padj
WBGene00002026	hsp-70	374.8473	24.086	1.00E-100	1.00E-100
WBGene00012121	T28C6.7	683.4814	23.3371	1.00E-100	1.00E-100
WBGene00017657	F21C10.7	406.7567	20.1661	1.00E-100	1.00E-100
WBGene00002016	hsp-16.2	856.3873	17.9534	1.00E-100	1.00E-100
WBGene00010369	chd-1	430.3263	15.0937	1.00E-100	1.00E-100
WBGene00002018	hsp-16.41	494.8661	14.4972	1.00E-100	1.00E-100
WBGene00001074	dpy-13	4705.2044	14.4111	1.00E-100	1.00E-100
WBGene00000402	cdh-10	280.2004	12.1438	1.00E-100	1.00E-100
WBGene00011696	eea-1	367.9106	12.0296	1.00E-100	1.00E-100
WBGene00010013	F54B3.1	969.1392	11.8977	1.00E-100	1.00E-100
WBGene00016577	clec-3	1662.4673	0.04775	1.00E-100	1.00E-100
WBGene00010123	F55G11.2	1058.1701	0.06253	1.00E-100	1.00E-100
WBGene00010745	dod-17	696.6498	0.1596	1.00E-100	1.00E-100
WBGene00001244	elo-6	4242.6399	0.4455	1.19E-85	2.19E-83
WBGene00007807	C29F3.7	789.0583	0.2653	1.115E-81	1.876E-79
WBGene00018343	F42A10.7	445.5492	0.2544	7.373E-76	1.032E-73
WBGene00016204	gsto-1	469.6329	0.2359	7.155E-72	8.868E-70
WBGene00019619	asp-14	5346.1094	0.209	1.22E-68	1.36E-66
WBGene00000003	aat-2	439.6023	0.2957	7.753E-67	8.22E-65
WBGene00016150	hint-3	649.6903	0.2768	3.153E-64	3.012E-62

Table S4. Summary of specific species of 14 ASVs.

ASV Number	Domain	Phylum	Class	Order	Family	Genus	Spiece
**ASV_2**	Bacteria	Pseudomonadota	Gammaproteobacteria	Moraxellales	Moraxellaceae	Acinetobacter	Acinetobacter sp.
**ASV_6**	Bacteria	Pseudomonadota	Gammaproteobacteria	Enterobacterales	Enterobacteriaceae	Escherichia	Escherichia coli
**ASV_13**	Bacteria	Bacillota	Bacilli	Lactobacillales	Enterococcaceae	Enterococcus	Enterococcus faecalis
**ASV_7**	Bacteria	Pseudomonadota	Gammaproteobacteria	Moraxellales	Moraxellaceae	Acinetobacter	Acinetobacter sp.
**ASV_22**	Bacteria	Bacillota	Clostridia	Eubacteriales	Lachnospiraceae	Blautia	Blautia wexlerae
**ASV_9**	Bacteria	Pseudomonadota	Gammaproteobacteria	Enterobacterales	Enterobacteriaceae	Escherichia	Escherichia coli
**ASV_15**	Bacteria	Actinomycetota	Actinomycetes	Micrococcales	Microbacteriaceae	Curtobacterium	Curtobacterium flaccumfaciens
**ASV_10**	Bacteria	Pseudomonadota	Gammaproteobacteria	Xanthomonadales	Xanthomonadaceae	Stenotrophomonas	Stenotrophomonas maltophilia
**ASV_4**	Bacteria	Pseudomonadota	Gammaproteobacteria	Enterobacterales	Erwiniaceae	Erwinia	Erwinia persicina
**ASV_3**	Bacteria	Pseudomonadota	Gammaproteobacteria	Enterobacterales	Enterobacteriaceae	Enterobacter	Enterobacter roggenkampii
**ASV_11**	Bacteria	Bacillota	Bacilli	Bacillales	Bacillaceae	Bacillus	Bacillus cereus
**ASV_8**	Bacteria	Pseudomonadota	Gammaproteobacteria	Enterobacterales	Enterobacteriaceae	Escherichia	Escherichia sp. UIWRF0665
**ASV_12**	Bacteria	Pseudomonadota	Gammaproteobacteria	Xanthomonadales	Xanthomonadaceae	Stenotrophomonas	Stenotrophomonas maltophilia
**ASV_21**	Bacteria	Bacillota	Bacilli	Bacillales	Paenibacillaceae	Paenibacillus	Paenibacillus sp. Baze28
